# Human dental pulp stem cells regulate allogeneic NK cells’ function via induction of anti‐inflammatory purinergic signalling in activated NK cells

**DOI:** 10.1111/cpr.12595

**Published:** 2019-04-05

**Authors:** Fei Yan, Ousheng Liu, Haixia Zhang, Yueying Zhou, Dian Zhou, Zekun Zhou, Yuhong He, Zhangui Tang, Songlin Wang

**Affiliations:** ^1^ Xiangya Stomatological Hospital and School of Stomatology Central South University Changsha Hunan China; ^2^ Beijing Key Laboratory of Tooth Regeneration and Function Reconstruction Capital Medical University School of Stomatology Beijing China; ^3^ Department of Oncology, The Second Xiangya Hospital Central South University Changsha Hunan China

**Keywords:** adenosine, CD73, dental pulp stem cells, immunoregulatory, natural killer cells, purinergic signalling

## Abstract

**Objectives:**

Mesenchymal stem cells (MSCs) could regulate the function of various immune cells. It remains unclear whether MSCs additionally possess immunostimulatory properties. We investigated the impact of human MSCs on the responsiveness of primary natural killer (NK) cells in terms of induction of anti‐inflammatory purinergic signalling.

**Material and Methods:**

We obtained human bone marrow mesenchymal stem cells (BMMSCs) and dental pulp stem cells (DPSCs). NK cells were isolated from peripheral blood of healthy volunteers. Activated NK cells were cultured with MSCs. Proliferation assay, apoptosis analysis, activating or inhibitory receptor expression and degranulation assay were used to explore NK cells’ function. High‐performance liquid chromatography was used to investigate the purinergic signalling in activated NK cells.

**Results:**

Both DPSCs and BMMSCs could impair proliferation and promote apoptosis of activated NK cells. Also, activated NK cells could cause DPSCs to lyse. Furthermore, the expression of activating NK cells’ receptors was decreased, but inhibitory receptors of NK cells were elevated following co‐cultivation. NK cells acquired CD73 expression, while MSCs could release ATP into the extracellular space where nucleotides were converted into adenosine (ADO) following co‐culture system. Under the existence of exogenous 2‐chloroadenosine (CADO), the cytotoxic capacity of NK cells was remarkably depressed in a concentration‐dependent manner.

**Conclusions:**

DPSCs and BMMSCs could depress NK cells’ function by hydrolysing ATP to ADO using CD39 and CD73 enzymatic activity. Our data suggested that DPSCs might represent a new strategy for treating immune‐related diseases by regulating previously unrecognized functions in innate immune responses.

## 1 INTRODUCTION

Adult stem cells exist in many tissues, including bone marrow, umbilical cord blood, placenta, liver, heart, lung and tendons.^1^, ^2^, ^3^, ^4^ By virtue of their low immunogenicity and immune‐regulating function, mesenchymal stem cells (MSCs) might be applied to modulate the immune responses in immune diseases, for example, graft‐vs‐host disease (GVHD) or autoimmune disease,^5^, ^6^ and regenerating damaged tissues, such as cardiac healing after myocardial infarction.^7^

Since bone marrow mesenchymal stem cells (BMMSCs) had low proliferative potential and were difficult to isolate from bone marrow through painful invasions,^8^ better sources of MSCs were needed for cell‐based clinical treatment.^9^ MSCs from dental tissues had self‐renewal capacity and multi‐differentiation potential and represented an easily available alternative source of cells.^10^ Dental pulp stem cells (DPSCs) were one of the most important stem cells from dental tissue because they had a superior proliferative potential compared to that of other types of stem cells from dental tissues. DPSCs also had low immunogenicity and immunoregulatory function.^11^ Our previous studies demonstrated that allogeneic DPSCs and periodontal ligament stem cells (PDLSCs) showed immunosuppressive effects on the activated T cells in vitro. In vivo, allogeneic PDLSCs were capable of regenerating and reconstructing the periodontal ligament attributed to periodontal disease, so that T‐cell immune function was lost. We also found that, via cell‐to‐cell contact, primarily mediated by programmed cell death 1 ligand and programmed cell death protein 1, allogenic PDLSCs were capable of suppressing B‐cell activation.^12^

Natural killer (NK) cells served as the main effector cells of innate immunity. In the bone marrow, NK cells evolved into progenitor cells, and they circulated as mature cells in the blood. They were critical for providing rapid responses to virally infected cells and in responding totumour formation.^13^ They had the characteristics of CD56 antigen surface expression and CD3‐negative expression, and their function was regulated mainly by activating and inhibiting the balance of signals transmitted by receptors interacting with specific HLA molecules on target cells.^14^ NK cells’ activation and interaction with target cells were caused by different receptors and co‐receptors. The natural cytotoxicity receptors (NCRs) NKp30, NKp44 and NKp46 were important receptors involved in NK cells’ activation and the mediating of cytotoxic activity.^15^, ^16^ Other crucial receptors for NK cells triggering were NKG2D and DNAM‐1.^17^, ^18^ Numbers of inhibitory receptors were presented by killer immunoglobulin‐like receptors (KIRs), and CD158 was one of the important inhibitory receptors.^19^ Normally, the expression of classical HLA or HLA‐E molecules on the surface of healthy autologous cells would prevent NK cells from triggering,^20^ because of these inhibitory receptors. Down‐regulated expression of HLA class I, or even the absence of a single HLA class I allele on the surface of a tumour or virally infected cells, might lead to the loss of inhibitory interaction and the activation of NK cells. In this case, the target cell was vulnerable to NK cell‐mediated killing. The interaction between MSCs from dental tissues and NK cells had not been fully explored. The present study analysed the effect of MSCs on NK cells correlation. We showed that MSCs were able to not only inhibit proliferation but also promote apoptosis of activated NK cells. However, activated NK cells could also efficiently lyse MSCs. After co‐cultured with MSCs, compared to NK cells cultured separately, the ability of NK cells for degranulation was significantly decreased. More importantly, we found that both BMMSCs and DPSCs could induce the functional expression of CD73 on NK cells, in a mechanism dependent of cell contact and soluble cytokines, promoting the activation of purinergic signalling. This information might have relevant implications for regulating NK cells’ activation through an autocrine or paracrine manner. This most likely was profound evidence in NK cells’ immunomodulation in the inflammatory microenvironment.

## 2 MATERIALS AND METHODS

### 2.1 Culture of human MSCs and K562 cells

All human stem cell experiments followed the ISSCR “Guidelines for the Conduct of Human Embryonic Stem Cell Research.” Human BMMSCs were kindly gifted from Professor Zhipeng Fan of Beijing Stomatological Hospital. The tissue of the dental pulp was cut from the extracted wisdom teeth, according to approved guidelines. The DPSCs were cultured according to previous methods.^10^ Briefly, the separated tissues were placed in a solution of 4 mg/mL of dispase (Roche Diagnostics Corp., Indianapolis, IN) and 3 mg/mL of collagenase type I (Worthington Biochemical Corp., Lakewood, NJ) for 1 hour at 37°C. The single‐cell suspensions were filtered using a 70 μm strainer (Falcon; BD Labware, Franklin Lakes, NJ). DPSCs were cultured in alpha DMEM and 15% foetal bovine serum (FBS; Gibco), 2 mmol/L of glutamine, 100 μg/mL of streptomycin and 100 U/mL of penicillin (Invitrogen) in an incubator under 5% CO_2_ at 37°C. DPSCs at passages 3‐5 were used in subsequent experiments. The flowcytometric analysis was used to characterize the expression of the typical surface of adherent cells in passage three by the absence of CD34, CD45, CD105, CD90 and CD73. The ability to differentiate into osteocytes, adipocytes and chondrocytes was confirmed (data not shown). K562 cells were obtained from the China Infrastructure of Cell Line Resource and were stored in RPMI‐1640 (GIBCO) with 10% FBS.

### 2.2 Isolation of NK cells

The heparin blood of healthy volunteers was diluted with an equal amount of phosphate buffer saline (PBS). Using RosetteSep (Stem Cell Technologies, Vancouver, BC, Canada), NK cells could be immediately isolated, following the instructions of the manufacturer. Using flowcytometry of CD56^+^CD3^‐^ lymphocytes, we assessed the purity of NK cells. We cultured the purified NK cells in RPMI 1640 (GIBCO), supplemented with 10% FBS (GIBCO), 40 ng/mL IL‐15 (R&D), 10 ng/mL IL‐12 (R&D) and 500 U/mL IL‐2 (Peprotech, Princeton, NJ, USA) to acquire activated NK cells.

### 2.3 Co‐culture of MSCs and NK cells

For apoptosis and degranulation experiments, we plated NK cells with 5:1 NK/MSCs ratio, which had been proven to be an appropriate ratio to illustrate that MSCs mediated inhibition of NK cell proliferation. Different MSC populations were isolated from different volunteers and correlated with NK cells. To analyse the involvement of adenosine (ADO) in the immunoregulatory phenomenon, a stable ADO analog, 2‐chloroadenosine (CADO), was used to detect its effect on activated NK cells. Analysis of 5’AMP and ADO was measured using chromatography with tandem mass spectrometry.

### 2.4 Monoclonal antibodies and flowcytometric analysis

The following monoclonal antibodies listed were applied and provided by BD Biosciences and Miltenyi Biotec: anti‐CD3‐FITC/CD16+CD56‐PE/CD45‐PerCP/CD19‐APC, anti‐CD73‐APC, anti‐CD107a‐APC, anti‐CD39‐APC, anti‐CD3‐PE, anti‐CD56‐APC, anti‐NKp44‐Viobright FITC, anti‐NKp30‐PE, anti‐CD3‐PerCP, anti‐NKG2D‐PE, anti‐CD158a‐FITC, anti‐CD158b‐PE, anti‐CD244‐FITC, anti‐DNAM‐1‐PE and anti‐CD69‐FITC. NK cells were analysed under a cytofluorometric process through trichromatic staining. Concisely, purification of NK cells was stained with fluorochrome‐conjugated MAbs, cultivated at 4°C for 30 minutes. Cells were then washed to remove the unbound reagents, and the cells were re‐suspended, studied by FACS Calibur. By gating for CD56^+^CD3^‐^ subsets cells, we evaluated the expression of different markers. In order to compare the surface density of NK receptors in NK cells cultured under different conditions, the mean fluorescence intensity (MFI) of selected mAb staining cells and unstaining cells (negative control) was calculated.

### 2.5 Proliferation assay

We labelled activated NK cells with 2 μmol/L carboxyfluorescein succinimidyl ester (CFSE; Thermo Fisher Scientific, Waltham, MA, USA). Next, we co‐cultured NK cells in flat‐bottomed 6‐well plates (Corning, Costar, MA), with plated DPSCs from allogeneic donors in a total volume of 2 mL RPMI‐1640 medium per well in the presence of IL‐15, IL‐12 and IL‐2 in triplicate at NK/MSCs ratios of 5:1. BMMSCs served as a positive control. Three days later, NK cells were collected and analysed by flowcytometry.

### 2.6 Apoptosis assay

By using the FITC Annexin V Apoptosis Detection kit Ⅱ (BD Biosciences) following the instructions of the manufacturer, we evaluated the percentage of apoptotic NK cells after 1 and 3 days cultured with or without DPSCs or BMMSCs, in the presence of IL‐2, IL‐12 and IL‐15.

### 2.7 NK cells’ degranulation assay

By using the frequency of degranulating NK cells by the expression of lysosome‐associated membrane protein‐1 (CD107a), the susceptibility of MSCs to NK cells lysis was evaluated.^21^ We treated isolated NK cells for 3 days with IL‐15, IL‐12 and IL‐2, and then incubated them for 5 hours at the NK/DPSCs ratios of 5:1 and 10:1 with differently expanded DPSCs. We added anti‐CD107a‐APC to the co‐cultures system promptly. After incubation for 1 hour, Golgistop reagent (BD Bioscience Pharmacology) was placed into incubation for 4 hours. Cells were then stained with a CD3/CD16+CD56/CD45/CD19 antibody (BD Biosciences) and analysed on a BD FACS Calibur. In another assay, we co‐cultured NK cells in flat‐bottomed 6‐well plates (Corning) with plated DPSCs from allogeneic donors in a total volume of 3 mL RPMI‐1640 medium per well in the presence of IL‐15, IL‐12 and IL‐2 at NK/MSCs ratios of 5:1 in triplicate. After 3 days, NK cells were collected and prepared for analysing degranulation at the NK/K562 ratios of 10:1. To detect the effect of CADO on NK cells, CADO was resolved into activated NK cells’ medium at the concentration of 10, 20 and 50 µM for 3 days, and the change of degranulation for CADO pre‐treated NK cells was detected.

### 2.8 ADO production and AMP utilization by NK cells’ assay

We cultured NK cells with pre‐seeded allogeneic BMMSCs or DPSCs at a ratio of 5:1 for 24 hours. We harvested and counted the NK cells by gentle pipetting and cultivated them in with 5'AMP as a substrate (at a concentration of 20 µM) for 30 minutes at 37°C in the incubator following the co‐culture. PBS with 5'AMP was also incubated at 37°C for 30 minutes as a cell‐free control to measure non‐enzymatic degradation of 5’‐AMP into ADO. All of the samples were then centrifuged for 3 minutes. After centrifugation, we transferred the supernatant fluid (PBS) to a 1.5 ml reaction tube. We added the same amount of an internal standard to each tube. After centrifuging for 10 minutes at 21 250 × *g* at 4°C, the supernatants were transferred into autosampler vials (with inserts). High‐performance liquid chromatography (HPLC) combined with tandem mass spectrometry was used to analyse supernatant, and its concentration was quantitatively analysed.

### 2.9 Statistical analysis

Prism 7 (GraphPad Software) was used for all statistical analysis. Comparisons were calculated by Student's unpaired *t* test, if two groups were assessed, or one‐way analysis of variance analysis (with Dunnett or Tukey post‐tests as indicated in figure legends) for more than two group comparisons. Levels of significance are shown as *P*‐values (**P < 0.01,*P < 0.05). Bar graphs represent mean ± SEM. Table 1 provides a selected core subset of the resourses in this paper. The entire list of 42 elements can be found as Supplementary Table S1 online

## 3 RESULTS

### 3.1 Effect of allogeneic MSCs on the bioactivity of activated NK cells

**Figure 1:**
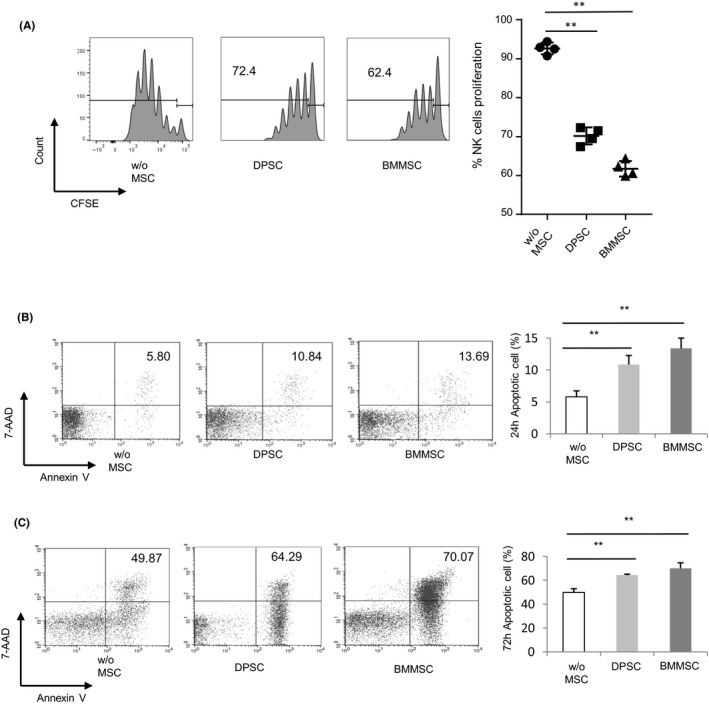
Effect of MSCs on proliferation and apoptosis of activated NK cells. A, Purified CFSE‐labelled NK cells (from peripheral blood from healthy donors) were co‐cultured for 3 d with allogeneic DPSCs and BMMSCs suspensions at 5:1 with IL‐2, IL‐12 and IL‐15. NK cell proliferation was assessed by CFSE analysis. Statistical analysis revealed that both DPSCs and BMMSCs inhibited NK cells’ proliferation. B, NK cells cultured for 24 h with or without DPSCs and BMMSCs (5:1) in the presence of stimuli were double stained with Annexin V and 7‐AAD. Statistical analysis showed that DPSCs and BMMSCs promoted NK cells’ apoptosis. C, NK cells cultured for 72 h with or without DPSCs and BMMSCs (5:1) in the presence of stimuli were double stained with Annexin V and 7‐AAD. Statistical analysis showed that DPSCs and BMMSCs promoted NK cells apoptosis. Results were shown as mean ± SEM, significance in (A‐C) was determined by one‐way analysis of variance followed by Dunnett post‐test (**P < 0.01)

As evaluated by flowcytometric analysis with a CD3/CD16+CD56/CD45/CD19 antibody, positively selected NK cells’ populations were in the range from 95% to 98% (Figure S1A). For the investigation of the inhibiting effect of allogeneic human DPSCs on activated NK cells, we cultured allogeneic pre‐seeded DPSCs with NK cells pre‐labelled with CFSE at the ratio of NK/MSCs 5:1 under the existence of exogenous IL‐2 (500 U/mL), IL‐12 (10 ng/mL) and IL‐15 (40 ng/mL), while BMMSCs co‐cultured with NK cells acted as positive control group. As shown in Figure 1A, the proliferation of NK cells was inhibited by BMMSCs and DPSCs.

For the investigation of the BMMSC‐mediated inhibition of NK cells’ proliferation or potential involvement of apoptosis in DPSCs, we stained the activated NK cells with or without DPSCs or BMMSCs at a 5:1 ratio for one and three days with Annexin V and 7‐AAD. Frequencies of Annexin V^+ ^apoptotic NK cells that were co‐cultured with DPSCs or BMMSCs were more than the group of which NK cells were cultured alone (Figure 1B). These data indicated that MSCs could impair the proliferation of NK cells, as well as promote apoptosis of NK cells.

### 3.2 Impact of MSCs on the cytotoxicity of activated NK cells

**Figure 2:**
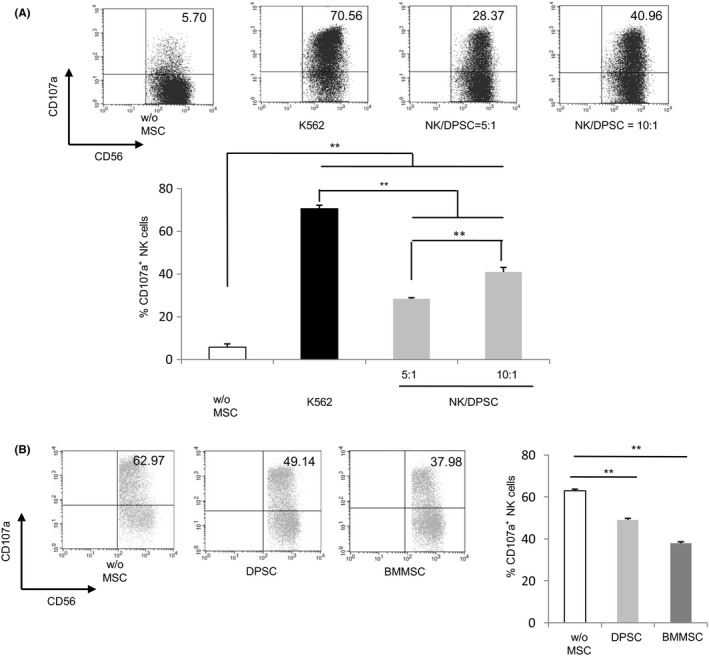
Effect of MSCs on cytotoxicity of activated NK cells. A, To quantify cytotoxic granule exocytosis, the surface expression of CD107a was analysed after activation of purified NK cells co‐cultured with target cells DPSCs (NK:Target) at 5:1 and 10:1 or NK‐susceptible target cell line (K562 cells). And it represented mean and SD of the percentage of CD107a on CD56‐positive cells in 5 different experiments. The percentage of gated NK cells expressing CD107a was determined as a measurement of degranulation. B, DPSCs and BMMSCs inhibited the cytotoxicity of activated NK cells. Freshly isolated allogeneic NK cells were co‐cultured at a ratio 5:1 in the presence or absence of DPSCs or BMMSCs for 72 h. Then, co‐cultured NK cells were collected for testing in a degranulation assay against K562 cells. The graph represents degranulation of NK cells cultured alone (w/o MSCs), degranulation of NK cells pre‐incubated with DPSCs and degranulation of NK cells pre‐incubated with BMMSCs. Results were shown as mean ± SEM, significance in (A) was determined by one‐way analysis of variance followed by the Tukey's test (**P < 0.01), significance in (B) was determined using the Dunnett test (**P < 0.01)

The cytotoxic degranulation capacity of NK cells by flowcytometry through the expression of CD107a (lysosomal‐associated membrane protein‐1, LAMP‐1) was evaluated. It was reported that MSCs owned immunogenicity and expressed ligands for activating NK cells’ receptors. Therefore, NK cell‐mediated killing of allogeneic DPSCs was investigated. DPSCs were co‐cultured with IL‐2/12/15‐activated NK cells at different ratios, and cytotoxic activity was measured. As a negative control, the CD107a expression of activated NK cells was quite low (5.7%; Figure 2A). However, the percentage of gated NK cells expressing CD107a was significantly increased after adding NK cells’ susceptible target cell line (K562 cells) (70.56%). Likewise, the percentage was significantly improved compared with the negative control group when NK cells co‐cultured with DPSCs at a 5 or 10 to 1 ratio (28.37%, 40.96%, respectively) (Figure 2A). It was shown that NK cells had a strong and dose‐dependent capacity to lyse DPSCs when cultured together.

Next, the cytotoxicity of NK cells after being co‐cultured with MSCs was investigated. We separated NK cells after cultivation with MSCs and exposed them to K562 cells. CD107a was strongly expressed on the cell surface of activated NK cells, but was lower when activated NK cells were pre‐incubated with DPSCs or BMMSCs (Figure 2B). Our result revealed that MSCs could depress the cytotoxicity of NK cells.

### 3.3 Effect of MSC on the expression of activating and inhibitory receptor of NK cells

**Figure 3:**
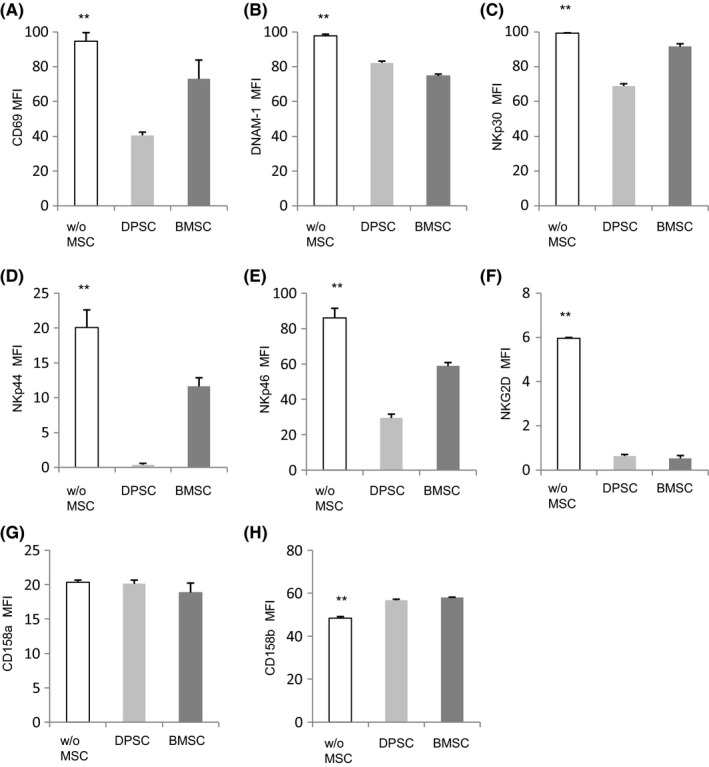
MSCs induce modification of surface molecules of NK cells. Purified NK cells (from peripheral blood from healthy donors) were co‐cultured with or without allogeneic DPSCs and BMMSCs suspensions at 5:1 with IL‐2, IL‐12 and IL‐15, and then stained for the surface expression of the activating marker CD69 (A), activating receptors DNAM‐1 (B), NKp30 (C), NKp44 (D), NKp46 (E) and NKG2D (F), including inhibitory receptor CD158a (G) and CD158b (H). Results were shown as mean ± SEM, significance determined by the Dunnett test (**P < 0.01)

Activated NK cells exhibited high expression of CD69, DNAM‐1, NKp30, NKp44, NKp46 and NKG2D, but significantly decreased following co‐culture with DPSCs and BMMSCs, respectively (Figure 3A‐F). On the other hand, inhibitory receptor expression of CD158a and CD158b was elevated following co‐culture with DPSCs and BMMSCs, respectively (Figure 3G, H). It showed that CD158b had a significant increase when NK cells interacted with MSCs.

### 3.4 Effect of MSCs on CD73 expression of NK cells

**Figure 4:**
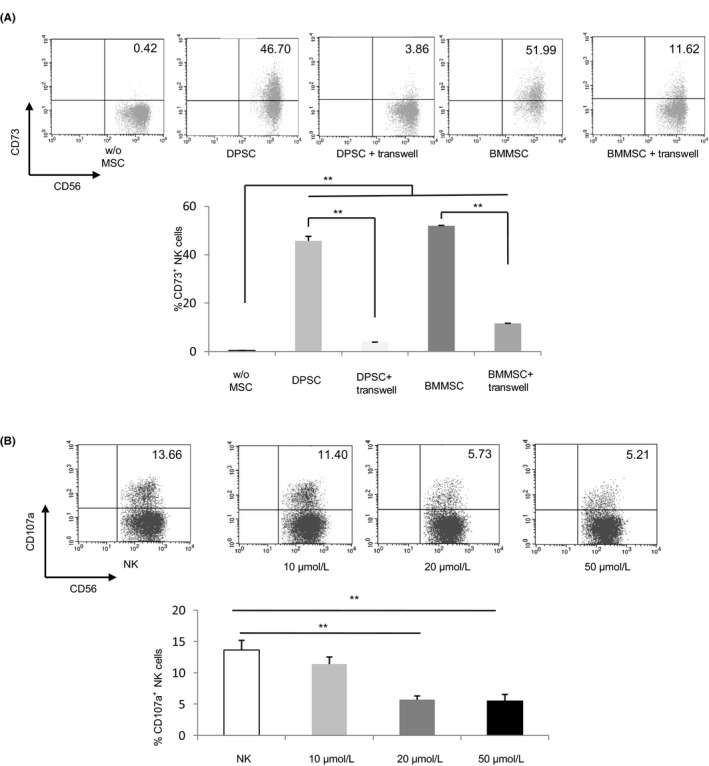
CD73 expression on NK cells and the effect of ADO analog on NK cells’ functions. A, DPSCs and BMMSCs increased CD73 expression of NK cells through contact‐dependent and independent mechanisms. Freshly isolated allogeneic NK cells were co‐cultured at a ratio 5:1 in the presence or absence of DPSCs or BMMSCs for 72 h in transwell or direct contact. These cells were subsequently sorted on the basis of CD56^+^CD3^−^CD73^+^ phenotype tested by flowcytometry. The graph represents CD73 expression of NK cells cultured alone (w/o MSCs), CD73 expression of NK cells pre‐incubated with DPSCs or BMMSCs through direct contact (DPSC or BMMSC), and CD73 expression of NK cells pre‐incubated with DPSCs or BMMSCs through transwell system (DPSC + transwell or BMMSC + transwell). B, NK cells were cultured with/without different concentration (10, 20 and 50 μmol/L) of CADO overnight, washed and further incubated with K562 cells for 4 h. CD107a surface expression was analysed on the NK cells as a readout for NK cell degranulative capacity. Significance in (A) was determined using the Tukey's test, significance in (B) was determined using the Dunnett test (**P < 0.01)

First, NK cells were coated in the upper cavity of the transwell system and physically separated from MSCs (at a 5:1 ratio, in the lower chamber). In cell contact experiments, NK cells were co‐cultured with DPSCs or BMMSCs. Generally, the percentage of CD73 expressing NK cells in peripheral blood was very low (0.42%); yet the expression rate of CD73 in NK cells of transwell and mixed co‐culture group was significantly higher than that of the control group (Figure 4A). Besides, we also found that the expression rate of CD73 was higher than that of the transwell system under intercellular contact conditions (Figure 4A).

### 3.5 Effect of exogenous CADO on cytotoxicity of activated NK cells

Once proved NK cells could acquire ectonucleotidase CD73 expression when co‐cultured with MSCs, we continued to detect the effect of ADO, the metabolized product of CD73, on anti‐tumour function. It became clear that when these CADO pre‐treated NK cells were exposed to K562 leukaemic cells, at a concentration of 20 μmol/L, they could significantly inhibit the expression of CD107a in comparison with NK cells cultured without CADO (Figure 4B).

### 3.6 Effect of exogenous CADO on the expression of activating and inhibitory receptor of NK cells

**Figure 5:**
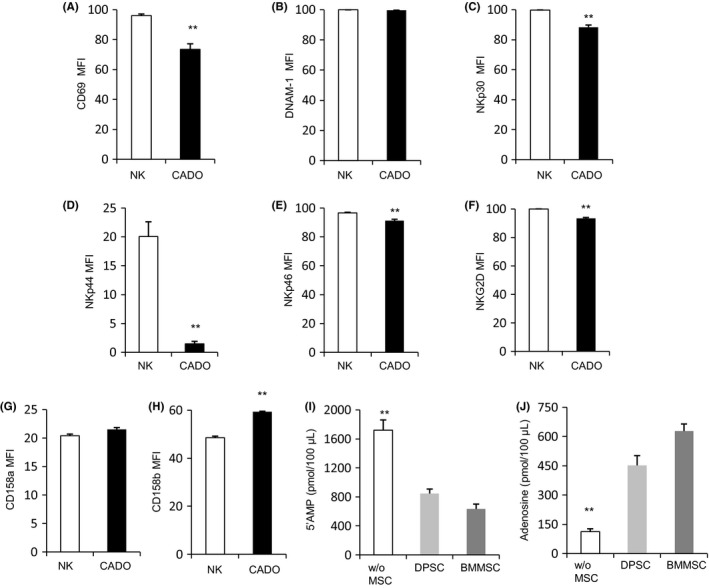
ADO induces modification of surface molecules of NK cells. A‐H, Purified NK cells (from peripheral blood from healthy donors) were co‐cultured with or without ADO at the concentration of 20 μmol/L and then stained for the surface expression of the activating marker CD69, activating receptors DNAM‐1, NKp30, NKp44, NKp46 and NKG2D, including inhibitory receptor CD158a and CD158b.(I, J) NK cells cultured with or without MSCs were cultivated in PBS with 5’AMP as a substrate for 30 min at 37°C. Cell‐free supernatants were collected and analysed using chromatography with tandem mass spectrometry for the levels of (I) residual 5′AMP (as a readout of substrate utilization) or (J) ADO (as a read out of product accumulation). Results were shown as mean ± SEM, significance in (A‐H) was determined by unpaired two‐tailed Student's *t* test (**P < 0.01), significance in (I, J) was determined using the Dunnett test (**P < 0.01)

When co‐cultured with MSCs, the reducing anti‐tumour ability of activated NK cells was due to the imbalance between an activating and inhibitory receptor. We hypothesized this phenomenon was also fit for the decreasing degranulation of NK cells pre‐treated with CADO. Activated NK cells showed decreased expression of CD69, NKp30, NKp44, NKp46 and NKG2D, but inhibitory receptor presented elevated expression in the presence of CADO (Figure 5A‐H).

## 4 DISCUSSION

Our data showed that MSCs could significantly inhibit the proliferation capacity of NK cells and increase the apoptosis potential of NK cells. In addition, NK cells could directly lyse DPSCs. However, MSCs showed an inhibitory effect on NK cell‐mediated cytotoxicity. We also found that NK cells interacting with MSCs could achieve CD73 expression on the cells surface and acquired the ability to convert 5’AMP to ADO. With the help of ADO, NK cell activation could be regulated in an autocrine or paracrine manner. These data might be relevant to MSC‐induced immunosuppression (eg to treat GVHD).

MSCshad been shown to inhibit the proliferation of activated T cells in vitro and in vivo. Human adult mesenchymal‐like progenitor cells derived from cardiac adipose tissue could suppress the alloproliferation of T cells in a dose‐dependent manner and modulate the secretion of proinflammatory cytokines (IL‐6, TNF‐α and IFN‐γ) specifically.^27^ In the present study, BMMSCs and DPSCs were observed to inhibit the proliferation of NK cells; this was similar to the most studies. Besides, compared with NK cells cultured alone, MSCs highly elevated NK cells’ apoptosis rate in co‐cultures, this finding was different from another scholar,^31^ and this might be due to the different cytokines and their concentration. Similarly to Spaggiar,^32^ MSCs could inhibit activated NK cells’ proliferation. Sotiropoulou found that MSCs inhibited IL‐15–induced NK cells’ proliferation both in contact and in transwell systems without inducing cell death,^33^ yet our data showed that MSCs could promote NK cell apoptosis. We speculated that the increased apoptosis of NK cells observed by MSCs could be due to the over‐stimulation of NK cells and induction of NK cells’ death.

In the present study, we analysed degranulation of NK cells to allogeneic DPSCs at a different ratio. The experiments showed that the percentage of NK cells expressing CD107a was very low without MSCs. Comparatively, the rate of NK cells to DPSCs was significantly higher than that of NK cells cultured separately. This study, combined with low levels of HLA class I molecules on the surface of DPSCs, resulted in low immunogenicity. Besides, HLA class I molecules’ low expression was conducive to NK‐mediated cracking of DPSCs.^34^

Another important issue associated with the NK cells and allogeneic MSCs interaction was whether the NK cells co‐cultured with MSCs could alter the anti‐tumour capacity of NK cells or not. In this consideration, it became apparent that the anti‐tumour function of NK cells was significantly decreased when interacting with MSCs, which supported the hypothesis that after interaction with heterogeneous MSCs, NK cells showed impaired anti‐tumour activity.^35^

The expression of CD69 on NK cells’ surface was analysed to identify NK cell activation since CD69 was an early activation marker that was closely related to the killing activity of NK cells. In this study, MSCs were capable of obviously inhibiting the activation of heterogeneous NK cells in vitro culture. In order to further study the reduction of anti‐tumour activity of NK cells, flowcytometry was used to analyse the surface molecular‐mediated inhibition signal and activation signal. The suppressor receptor matched the HLA class I molecule and activated the receptor to recognize specific ligands on the surface of the target cell. It was noteworthy that by using monoclonal antibody, most of the activating receptors on the surface of NK cells, such as NKG2D, DNAM‐1, NKp46, NKp44 and NKp30, were significantly decreased after interaction with MSCs. It was proved that NKp30, NKG2D and DNAM‐1 were the most important NK cells activating receptors involved in the anti‐tumour function. Besides, one of the inhibitory receptors on NK cells’ surface, CD158b, was also remarkably increased after co‐cultured with MSCs, while CD158a did not affect by interaction with MSCs. The activating signals were decreased, but inhibitory receptors were increased. The anti‐tumour activity of NK cells was impaired.

To deeply understand the mechanisms of NK cells and MSCs interaction, we found a new theory issued by Chatterjee^42^ and it was found that NK cells acquired ectonucleotidase CD73 expression upon exposure to BMMSCs. In our study, it was also discovered that NK cells could own CD73 expression after interaction with BMMSCs or DPSCs. Recently, Monguió‐Tortajada found that MSC co‐culture lead to sustained CD73 expression and to active 5′AMP hydrolysis by monocytes, in a mechanism dependent of cell contact. In addition, the local treatment with MSCs promoted the CD73 expression on host monocytes in a swine model of myocardial infarction, and the upregulation of CD73 on MSC‐conditioned monocytes was an effective mechanism to amplify the long‐lasting immunomodulatory and healing effects of MSCs delivery.^43^ We consistently showed that the expression of CD73 on NK cells co‐cultured in contact with DPSCs or BMMSCs had significant differences compared with transwell co‐culture system. Moreover, the date also demonstrated that NK cells co‐cultured with DPSCs and BMMSCs in the transwell system exerted higher CD73 expression in comparison with the control value. These results revealed that soluble factors released by DPSCs or BMMSCs might be potent enough to modulate NK cells’ phenotype. This finding was distinct from another scholar^43^; this might be due to the different source of stem cells and immune cells.

For ectonucleotidase CD73‐positive expression of NK cells co‐cultured with MSCs, we inferred that NK cells could catalyse ATP into AMP by means of CD39‐positive expression (Figure S2A) and then acquired the ability to convert AMP into ADO. It was clear that ADO covered by acquired CD73 on the surface of NK cells when interacted with MSCs could impair NK cells’ functions. Recently, multiple reports suggested that the loss function of ADO production was at least in part related to the onset of autoimmune disorders (ie multiple sclerosis, encephalomyelitis, rheumatoid arthritis, diabetes and uveitis).^43^ More importantly, locally ADO production played a crucial role in stimulating the release of anti‐inflammatory cytokines (ie IL‐10) and inhibiting the release of proinflammatory molecules (ie TNF‐α and nitric oxide) in animal models of inflammation (ie myocardial infarction). It was also accepted that ADO could suppress inflammation by regulating various immune cells, such as monotype, macrophage, dendritic cells, B and T cells. We also investigated the effect of ADO on NK cells’ functions. Due to the metabolic instability of ADO, the use of ADO was frequently superseded by the use of its metabolically stable analog CADO, and CADO was dissolved into the NK cells medium. Through degranulation assay of NK cells, we found that CADO, at a concentration of 20 μmol/L, was the minimal effective concentration to inhibit the anti‐tumour function of NK cells, and this concentration was used for the next experiment.

The effect of CADO at the concentration of 20 μmol/L on early activation marker CD69 and other activating and inhibitory receptors of NK cells was measured by flowcytometry. In this study, it was found that CD69 and activating receptors NKp30, NKp44, NKp46 and NKG2D were significantly decreased, and inhibitory receptor CD158b had a noticeable increase. This result was nearly the same as the conclusion of NK cells and MSCs interaction. Work by Wang also showed that adenosine leads to the downregulation of activating receptors on NK cells when stimulated with cytokines.^46^

Finally, with the help of mass spectroscopy, it was illustrated that acquired ectonucleotidase CD73 on NK cells’ surface was accompanied by increased enzyme activity. First, it was shown that the CD73 substrate, 5′‐AMP utilization, was increased by NK cells interacting with pre‐seeded DPSCs. Second, it was directly documented that NK cells co‐cultured with DPSCs were able to produce a markedly higher concentration of ADO than NK cells cultured alone (Figure 5B).

In conclusion, by acting at multiple levels, for example, proliferation, apoptosis and degranulation capacity, both allogeneic DPSCs and BMMSCs had the ability to regulate the function of NK cells. NK cells interacting with MSCs might acquire the expression of external nucleotide CD73. These CD73‐positive NK cells had the ability to regulate the function of NK cells in either an autocrine or paracrine manner. This might be of great significance to immune regulation in the inflammatory microenvironment. Therefore, regulating the function of NK cells and other cells further supported that heterogeneous MSCs might be a promising method to treat several inflammatory disease states.

## CONFLICTS OF INTEREST

The authors declare that there are no conflicts of interest regarding the publication of this paper. The data used to support the findings of this study are available from the corresponding author upon request.
