# Supplementary material for: Human dental pulp stem cells regulate allogeneic NK cells’ function via induction of anti‐inflammatory purinergic signalling in activated NK cells
Source: Cell Prolif. 2019 Apr 5;52(3):e12595. doi: 10.1111/cpr.12595 (PMC6536423; doi:10.1111/cpr.12595)
Supplement: Supplementary file 3 [file CPR-52-e12595-s003.docx]

KEY RESOURCES TABLE

| REAGENT or RESOURCE | SOURCE | IDENTIFIER |
| --- | --- | --- |
| Antibodies |  |  |
| APC Mouse anti-Human CD73 | BD Biosciences | Cat# 560847 |
| APC Mouse Anti-Human CD107a | BD Biosciences | Cat# 560664 |
| APC Mouse IgG1 κ Isotype Control | BD Biosciences | Cat# 554681 |
| APC Mouse anti-Human CD39 | BD Biosciences | Cat# 560239 |
| APC Mouse IgG2b κ Isotype Control | BD Biosciences | Cat# 555745 |
| 7-AAD | BD Biosciences | Cat# 559925 |
| PE Mouse Anti-Human CD3 | BD Biosciences | Cat# 555340 |
| APC Mouse Anti-Human CD56 | BD Biosciences | Cat# 341025 |
| Multitest™ CD3/CD16+CD56/CD45/CD19 | BD Biosciences | Cat# 342416 |
| CD337 (NKp30)-PE | Miltenyi Biotec | Cat# 130-118-542 |
| CD336 (NKp44)-VioBright FITC | Miltenyi Biotec | Cat# 130-118-542 |
| CD3-PerCP | Miltenyi Biotec | Cat# 130-113-131 |
| CD335 (NKp46)-VioBright FITC | Miltenyi Biotec | Cat# 130-104-518 |
| CD314 (NKG2D)-PE | Miltenyi Biotec | Cat# 130-092-672 |
| CD158a/h (KIR2DL1/DS1)-FITC | Miltenyi Biotec | Cat# 130-118-973 |
| CD158b (KIR2DL2/DL3)-PE | Miltenyi Biotec | Cat# 130-092-618 |
| CD244 (2B4)-FITC | Miltenyi Biotec | Cat# 130-099-039 |
| CD226 (DNAM-1)-PE | Miltenyi Biotec | Cat# 130-092-476 |
| CD69-FITC | Miltenyi Biotec | Cat# 130-113-523 |
| CD132-PE | Miltenyi Biotec | Cat# 130-104-587 |
| [Anti-IFN-γ-FITC](https://www.miltenyibiotec.com/US-en/products/macs-flow-cytometry/antibodies/primary-antibodies/anti-ifn-g-antibodies-human-45-15-1-50.html#fitc:for-100-tests) | Miltenyi Biotec | Cat# 130-113-492 |
| Anti-TNF-α-PE | Miltenyi Biotec | Cat# 130-091-651 |
| Biological Samples |  |  |
| Human PBMC | The Beijing Stomatological Hospital of Capital Medical University |  |
| Extracted Wisdom Teeth | The Beijing Stomatological Hospital of Capital Medical University |  |
| Chemicals and Recombinant Proteins |  |  |
| RosetteSep™ Human NK Cell Enrichment Cocktail | StemCell | Cat# 15065 |
| BD GolgiStop^TM^ | BD Biosciences | Cat# 554724 |
| CytoTox96 non-radioactive cytotoxicity kit | Promega | Cat# G1780 |
| CellTrace™ CFSE Cell Proliferation Kit | Thermo Fisher Scientiﬁc | Cat# C34554 |
| [Monensin sodium](https://www.sigmaaldrich.com/catalog/product/usp/1445481?lang=zh&region=CN) | Sigma Aldrich | Cat# 1445481 |
| [Histopaque®-1077](https://www.sigmaaldrich.com/catalog/product/sigma/10771?lang=zh&region=CN) | Sigma Aldrich | Cat# 10771 |
| Recombinant Human IL-15 | R&D Systems | Cat# 247-IL-005 |
| Recombinant Human IL-12 | R&D Systems | Cat# 219-IL-005 |
| Recombinant Human IL-2 | PeproTech | Cat# 200-02A |
| Recombinant Human IL-18 | R&D Systems | Cat#U77776 |
| *Continued* |  |  |
| REAGENT or RESOURCE | SOURCE | IDENTIFIER |
| CADO | Sigma Aldrich | Cat# C5134 |
| [Pharmingen™ Leukocyte Activation Cocktail, with GolgiPlug™](http://www.bdbiosciences.com/cn/reagents/research/antibodies-buffers/immunology-reagents/immunology-buffers-and-ancillary-reagents/leukocyte-activation-cocktail-with-bd-golgiplug/p/550583) | BD Biosciences | Cat# 550583 |
| 5′-AMP | Sigma Aldrich | Cat# C01930 |
| Critical Commercial Assays |  |  |
| IntraSure™ Kit | BD Biosciences | Cat# 641776 |
| FITC Annexin V Apoptosis Detection Kit II | BD Biosciences | Cat# 556570 |
| Experimental Models: Cell Lines |  |  |
| K562 | China Infrastructure of Cell Line Resource | Cat# 3111C0001CCC000039 |
| Software and Algorithms |  |  |
| FlowJo 9 software | FlowJo, LLC | https://www.ﬂowjo.com,  RRID:SCR_008520 |
| GraphPad Prism 7 software | GraphPad Software | https://www.graphpad.com,  RRID:SCR_002798 |
